# Presentation Delay After Symptom Recognition Among Breast Cancer Patients in Mongolia: A Retrospective Study

**DOI:** 10.3390/healthcare14193270

**Published:** 2026-10-02

**Authors:** Bolormaa Dalkhjav, Avirmed Damdin, Naranchimeg Sodovsuren, Solongo Dashnyam

**Affiliations:** 1 Department of Adult Health Nursing, School of Nursing, Mongolian National University of Medical Sciences (MNUMS), Ulaanbaatar 14210, Mongolia; bolor3439@gmail.com or amd13f014@gt.mnums.edu.mn; 2 Department of Obstetrics and Gynecology, Ach Medical University, Ulaanbaatar 18101, Mongolia; avirmed@ach.edu.mn; 3 Department of Communication Skills, School of Biomedicine, Mongolian National University of Medical Sciences (MNUMS), Ulaanbaatar 14210, Mongolia; naranchimeg@mnums.edu.mn

**Keywords:** breast cancer, presentation delay, breast symptoms, advanced-stage disease, delayed presentation, Mongolia

## Abstract

**Highlights:**

**What are the main findings?**

- Presentation delay of ≥1 month occurred in 69.3% of symptomatic breast cancer patients, and the initial presenting symptom was the principal factor associated with delay.
- Presentation delay was strongly associated with advanced-stage (III–IV) breast cancer, with approximately fourfold higher adjusted odds of advanced-stage disease (aOR 4.48, 95% CI 2.12–9.49).

**What are the implications of the main findings?**

- Improving recognition of breast symptoms beyond the classic breast lump may help encourage earlier medical consultation among symptomatic women.
- Strategies to reduce the interval between symptom recognition and first medical consultation may contribute to earlier-stage breast cancer diagnosis in Mongolia.

**Abstract:**

**Background/Objectives:** Delayed medical consultation after recognizing breast symptoms may contribute to breast cancer being diagnosed at a more advanced stage. Evidence on presentation delay and its determinants remains limited in Mongolia. This study aimed to determine the frequency and factors associated with presentation delay among symptomatic breast cancer patients and to examine its association with tumor characteristics at diagnosis. **Methods:** This retrospective observational study included symptomatic patients with newly diagnosed breast cancer treated at the National Cancer Center in Ulaanbaatar, Mongolia, between 2022 and 2024. Presentation delay was defined as ≥1 month from recognition of the initial breast symptom to first medical consultation. Sociodemographic, clinical, and tumor characteristics were compared according to presentation delay. Multivariable logistic regression was used to identify factors associated with presentation delay and to assess its association with advanced-stage disease. **Results:** Overall, 169 (69.3%) patients had a presentation delay of ≥1 month. Initial presenting symptom was independently associated with presentation delay. Compared with a breast lump, a long-standing breast lump (adjusted odds ratio [aOR] 7.92, 95% confidence interval [CI] 3.14–19.98), breast pain (aOR 3.80, 95% CI 1.89–7.66), and nipple changes (aOR 3.32, 95% CI 1.20–9.21) were associated with higher odds of presentation delay. Age, marital status, education, residence, and body mass index were not independently associated with delay. Stage III–IV disease was more frequent among patients with presentation delay than among those presenting within one month (39.1% vs. 13.3%), and presentation delay was independently associated with higher odds of stage III–IV disease (aOR 4.48, 95% CI 2.12–9.49). **Conclusions:** Presentation delay was common among symptomatic breast cancer patients in Mongolia. Among the factors measured in this study, initial presenting symptom showed the strongest association with presentation delay, while presentation delay was also associated with advanced-stage disease at diagnosis.

## 1. Introduction

Breast cancer is one of the most commonly diagnosed cancers worldwide and remains a major cause of cancer-related morbidity and mortality among women. Globally, female breast cancer accounted for approximately 2.3 million new cases and 11.6% of all newly diagnosed cancers in 2022 [1]. Although breast cancer incidence in Mongolia remains relatively low compared with many other countries, its burden has increased over time. GLOBOCAN 2022 estimated 227 new breast cancer cases in Mongolia, accounting for 7.1% of all cancers diagnosed among women, with an age-standardized incidence rate of 12.6 per 100,000 women [2]. Earlier Mongolian registry data analyzed by Shreves et al. also demonstrated a continuing increase in breast cancer incidence between 1998 and 2014, with higher incidence among urban than rural women [2]. These trends underscore the growing relevance of breast cancer early detection in Mongolia. Although advances in early detection, diagnosis, and treatment have improved outcomes, substantial disparities persist between countries and healthcare settings, particularly in low- and middle-income countries (LMICs) [1,3,4]. The World Health Organization (WHO) Global Breast Cancer Initiative identifies health promotion for early detection, timely diagnosis, and comprehensive breast cancer management as three key pillars for reducing breast cancer mortality [5].

For women with symptomatic breast cancer, timely diagnosis depends partly on the interval between recognizing a breast symptom and seeking medical attention. The Aarhus Statement provides a standardized framework for research on pathways to cancer diagnosis and recommends distinguishing the patient interval from subsequent healthcare-related diagnostic intervals [6]. In the context of symptomatic breast cancer, the patient interval refers to the period between a patient’s recognition of an initial breast symptom and first consultation with a healthcare professional [6]. However, studies of breast cancer have used different definitions and thresholds for delayed presentation, including intervals of one, two, or three months, which complicates comparisons across populations [7,8].

Presentation delay among women with breast cancer is multifactorial. Systematic reviews from LMICs have identified limited awareness of breast cancer symptoms, failure to perceive symptoms as serious, fear of cancer diagnosis or treatment, socioeconomic disadvantage, limited social support, geographical barriers, and difficulties accessing healthcare as potential contributors to delayed help-seeking [7,8,9]. Associations with sociodemographic characteristics such as age, marital status, education, and place of residence have been less consistent across populations, suggesting that determinants of presentation delay may be strongly influenced by local sociocultural and healthcare contexts [7,8].

The nature of the initial breast symptom may be particularly important in determining when women seek medical care. Although a breast lump is the most widely recognized symptom of breast cancer, breast cancer can present with breast pain, nipple abnormalities, skin changes, and other non-lump symptoms [10]. In a national study of symptomatic breast cancer patients, women presenting with non-lump symptoms had longer patient intervals than those presenting with a breast lump alone; the proportion waiting ≥90 days was 15% among women with non-lump symptoms compared with 7% among those with a breast lump alone [10]. Qualitative evidence also indicates that symptom interpretation, monitoring of initially mild or painless changes, fear, and beliefs about cancer can influence the decision to seek medical attention [11]. These findings suggest that awareness strategies focused primarily on breast lumps may insufficiently address the broader spectrum of symptomatic breast cancer.

The consequences of prolonged presentation are clinically important. Longer intervals before medical consultation may postpone diagnostic assessment and potentially contribute to diagnosis at a more advanced stage, when treatment is generally more complex and prognosis is poorer [5,6]. Nevertheless, the relationship between presentation delay and stage at diagnosis is not necessarily straightforward. Tumor biology and growth rate, the nature of the presenting symptom, and subsequent healthcare-system intervals may also influence disease stage at diagnosis [6,8]. Therefore, the association between the patient interval and tumor characteristics should be evaluated within individual healthcare settings rather than assuming that advanced-stage disease is solely attributable to delayed help-seeking.

This issue is particularly relevant in Mongolia. Although historically reported breast cancer incidence has been relatively low compared with many other countries, the burden of breast cancer has increased and has been recognized as an important emerging health policy issue [12]. Earlier work on breast cancer in Mongolia highlighted challenges related to late-stage diagnosis, limited early-detection infrastructure, geographical access, and the centralized organization of cancer services [12]. More recent initiatives have also emphasized the need to strengthen multidisciplinary breast cancer care in Mongolia [13]. In previous Mongolian data, breast lumps were a predominant reason for seeking medical attention, while both patient- and healthcare-provider-related factors were reported as contributors to delayed diagnosis. However, contemporary evidence specifically examining the interval between patient recognition of an initial breast symptom and first medical consultation, its independent determinants, and its relationship with tumor stage remains limited in Mongolia.

A better understanding of presentation delay in the Mongolian context may help identify modifiable factors that can be addressed through breast health education and early-diagnosis strategies. In particular, determining whether delay is predominantly associated with sociodemographic characteristics or with the nature of the presenting symptom could help refine the content and targeting of public awareness interventions. Accordingly, this study aimed to determine the frequency of presentation delay, defined as a patient interval of ≥1 month from recognition of the initial breast symptom to first medical consultation, among symptomatic breast cancer patients in Mongolia. We further aimed to identify sociodemographic and symptom-related factors associated with presentation delay and to examine its association with tumor characteristics at diagnosis.

## 2. Materials and Methods

### 2.1. Study Design and Setting

This retrospective observational study was conducted at the National Cancer Center in Ulaanbaatar, Mongolia. Medical records of patients with breast cancer treated between 2022 and 2024 were reviewed. The National Cancer Center is the principal tertiary referral center for cancer diagnosis and treatment in Mongolia and receives patients from Ulaanbaatar and other regions of the country. Breast cancer evaluation in Mongolia may involve initial assessment in primary or secondary healthcare facilities followed by referral for specialist evaluation when malignancy is suspected, while the National Cancer Center provides specialized diagnostic and oncological services. Patients may also enter the diagnostic pathway through early-detection or other health examinations. The present study, however, was restricted to patients with self-recognized breast symptoms in order to specifically evaluate presentation delay before first medical consultation.

The study focused on the interval between patients’ recognition of their initial breast symptom and their first medical consultation and on the relationship between this interval and clinical characteristics at diagnosis. The terminology for diagnostic intervals was based on the conceptual framework of the Aarhus Statement [6].

The study was conducted in accordance with the Declaration of Helsinki. Ethical approval was obtained from the Research Ethics Review Committee of the Mongolian National University of Medical Sciences on 18 November 2022 (Approval No. 2022/3-10).

### 2.2. Study Population and Participant Selection

A total of 564 breast cancer records from 2022–2024 were initially identified. Patients were eligible if they were female, had a first diagnosis of breast cancer, had histopathologically confirmed malignant breast disease, and had not previously received treatment for recurrent breast cancer. Male patients, patients with recurrent or previously treated breast cancer, and those whose pathological examination did not confirm breast malignancy were excluded.

Following these exclusions, 343 patients were eligible for further assessment. Because the primary objective of the present study was to examine presentation delay after recognition of breast symptoms, patients whose breast cancer was detected in the absence of a patient-recognized breast symptom, including detection through screening, routine examination, or other circumstances, were excluded from the primary analysis. The final analytic sample therefore comprised 244 symptomatic patients with breast cancer (Figure 1).

### 2.3. Data Collection and Study Variables

Data were obtained from available medical records and study records using a structured data collection form. Information collected included sociodemographic characteristics, clinical characteristics, initial breast symptoms, tumor characteristics, and the time between recognition of the initial breast symptom and first medical consultation.

Information on the timing of initial symptom recognition and first medical consultation was obtained retrospectively from the available medical and study records and therefore reflected patient-reported information as documented in these records. The patient interval was available in predefined categories (<1 month, 2–3 months, 4–12 months, 1–9 years, and >10 years), rather than as exact dates. Because the analysis used these recorded categories, exact dates could not be independently reconstructed or verified, particularly for patients reporting prolonged intervals.

Sociodemographic variables included age, marital status, education level, and place of residence. Body mass index (BMI) was calculated as weight in kilograms divided by height in meters squared (kg/m^2^) and categorized as <25 and ≥25 kg/m^2^ for the present analysis.

Clinical and tumor-related variables included the initial presenting breast symptom, tumor size, cancer stage, histological type, and molecular/immunohistochemical (IHC) subtype. Initial presenting breast symptoms were classified as breast lump, long-standing breast lump, breast pain, or nipple changes based on information documented in the available medical and study records. In the source data, a ‘long-standing breast lump’ referred to a lump described in the recorded clinical history as having initially been small or painless and subsequently becoming more noticeable, including enlargement or development of pain. This category reflected the documented clinical description of the presenting symptom and was not defined according to the ≥1-month threshold used to classify presentation delay. Tumor size was categorized as <5 cm or ≥5 cm. Cancer stage was recorded as in situ and stages I–IV. For analyses of advanced-stage disease, stages III and IV were combined and compared with earlier-stage disease.

### 2.4. Definition of Presentation Delay

The primary outcome was presentation delay, defined as a patient interval of ≥1 month from recognition of the initial breast symptom to first medical consultation. Patients who sought medical consultation within <1 month were classified as having no presentation delay, whereas those who first consulted a healthcare professional ≥1 month after symptom recognition were classified as having presentation delay. The ≥1-month threshold was selected to identify relatively early postponement of medical consultation after symptom recognition and has been used in previous breast cancer studies, although thresholds vary across the literature. This definition characterizes delay occurring before entry into the healthcare system and distinguishes it from subsequent diagnostic and treatment intervals, consistent with the conceptual framework for pathways to cancer diagnosis described in the Aarhus Statement [6].

For descriptive purposes, the original time-to-consultation categories were retained to illustrate the full distribution of the patient interval, ranging from <1 month to prolonged intervals of several years (Figure 2). To assess the robustness of the findings to the choice of cut-off, a sensitivity analysis using a more stringent definition of presentation delay was also performed.

### 2.5. Statistical Analysis

Categorical variables were summarized as frequencies and percentages. Participant characteristics were compared between patients who sought medical consultation within <1 month and those with a presentation delay of ≥1 month. Pearson’s chi-square test was used to assess associations between categorical variables; Fisher’s exact test was used when appropriate.

Binary logistic regression analyses were performed to identify factors associated with presentation delay. Presentation delay (≥1 month) was coded as the outcome, with presentation within <1 month as the reference outcome. Crude odds ratios (ORs) and 95% confidence intervals (CIs) were first estimated. A multivariable logistic regression model was then fitted including age group, marital status, education level, residence, BMI category, and initial presenting breast symptom. These variables represented the sociodemographic, anthropometric, and symptom-related factors available in the dataset that were considered potentially relevant to presentation delay. Adjusted odds ratios (aORs) with 95% CIs were reported. Overall effects of categorical variables were evaluated using Wald tests. Model calibration was assessed using the Hosmer–Lemeshow goodness-of-fit test, and Nagelkerke R^2^ was reported as an indicator of model explanatory performance.

Secondary logistic regression analyses examined whether presentation delay was associated with tumor characteristics at diagnosis. Separate models were fitted for advanced-stage disease (stage III–IV) and tumor size ≥5 cm. These models were adjusted for age, residence, and education, which were selected as potential sociodemographic confounders of the association between presentation delay and tumor characteristics. A parsimonious adjustment set was used for the secondary analyses to limit model complexity.

Exploratory interaction analyses were additionally performed to assess whether the association between initial presenting symptom and presentation delay differed according to residence or education. Interaction terms for initial symptom × residence and initial symptom × education were evaluated in separate multivariable logistic regression models. Because some interaction strata contained relatively small numbers, these analyses were considered exploratory.

As a sensitivity analysis, we examined a more stringent definition of presentation delay. Because the original patient-interval variable categorized time to first medical consultation as <1 month, 2–3 months, 4–12 months, 1–9 years, and >10 years, an exact ≥3-month threshold could not be reconstructed. We therefore used ≥4 months as the closest conservative alternative threshold and repeated the primary multivariable logistic regression using the same covariates.

All statistical tests were two-sided, and a *p*-value < 0.05 was considered statistically significant. Statistical analyses were performed using IBM SPSS Statistics, version 26.0 (IBM Corp., Armonk, NY, USA).

## 3. Results

### 3.1. Participant Characteristics and Presentation Delay

The primary analysis included 244 symptomatic patients with breast cancer. Of these, 75 (30.7%) sought medical consultation within one month of recognizing their initial breast symptom, whereas 169 (69.3%) had a presentation delay of ≥1 month. Participant characteristics according to presentation delay are presented in Table 1.

There were no statistically significant differences between patients with and without presentation delay according to age group (*p* = 0.424), marital status (*p* = 0.593), education level (*p* = 0.864), residence (*p* = 0.597), or BMI category (*p* = 0.743).

### 3.2. Clinical and Tumor Characteristics According to Presentation Delay

Clinical and tumor characteristics according to presentation delay are shown in Table 2. The distribution of initial presenting breast symptoms differed significantly between the two groups (*p* < 0.001). Among patients presenting within one month, a breast lump was the most frequent initial symptom (57.3%), compared with 24.9% among patients with presentation delay. In contrast, a long-standing breast lump was reported by 28.4% of patients with presentation delay compared with 9.3% of those presenting within one month. Breast pain was also more frequent among delayed presenters (36.1% vs. 24.0%).

Tumor stage differed significantly according to presentation delay (*p* = 0.003). Stage III–IV disease was present in 66 of 169 (39.1%) patients with presentation delay compared with 10 of 75 (13.3%) patients presenting within one month. Tumors ≥ 5 cm were also more frequent among delayed presenters (13.0% vs. 5.3%), although the overall difference in tumor-size category did not reach statistical significance (*p* = 0.073). Histological type (*p* = 0.749) and molecular/IHC subtype (*p* = 0.887) did not differ significantly according to presentation delay.

### 3.3. Factors Associated with Presentation Delay

After multivariable adjustment, initial presenting symptom remained significantly associated with presentation delay (*p* < 0.001). Compared with breast lump, long-standing breast lump was associated with higher odds of presentation delay (aOR 7.92, 95% CI 3.14–19.98; *p* < 0.001), as was breast pain (aOR 3.80, 95% CI 1.89–7.66; *p* < 0.001). Nipple changes were also associated with higher odds of presentation delay (aOR 3.32, 95% CI 1.20–9.21; *p* = 0.021) (Table 3).

Age, marital status, education, residence, and BMI were not independently associated with presentation delay in the adjusted model. The multivariable model was statistically significant overall (likelihood-ratio χ^2^ = 31.514, df = 10, *p* < 0.001) and showed acceptable calibration (Hosmer–Lemeshow *p* = 0.436; Nagelkerke R^2^ = 0.171).

### 3.4. Distribution of Time to First Medical Consultation

Although the primary analyses dichotomized presentation time at one month, the original waiting-time categories showed considerable variation in the duration before patients first sought medical consultation (Figure 2). The figure presents the full distribution from <1 month through ≥10 years, with the ≥1-month threshold indicating the definition of presentation delay used in the primary analyses.

### 3.5. Presentation Delay and Tumor Characteristics at Diagnosis

In a secondary analysis, we examined whether presentation delay was associated with tumor characteristics at diagnosis (Table 4). Presentation delay was strongly associated with advanced-stage disease. Patients with presentation delay had approximately fourfold greater crude odds of stage III–IV disease (OR 4.17, 95% CI 2.00–8.68; *p* < 0.001). The association remained after adjustment for age, residence, and education (aOR 4.48, 95% CI 2.12–9.49; *p* < 0.001).

The corresponding association with tumor size ≥ 5 cm was weaker and did not reach statistical significance (crude OR 2.66, 95% CI 0.88–8.00; *p* = 0.082; adjusted OR 2.59, 95% CI 0.83–8.08; *p* = 0.100).

### 3.6. Additional Analyses

The relationship between initial presenting symptom and presentation delay did not appear to differ significantly according to residence or education. There was no statistical evidence of an initial symptom × residence interaction (χ^2^ = 2.692, df = 3, *p* = 0.442) or an initial symptom × education interaction (χ^2^ = 3.547, df = 6, *p* = 0.738) (Supplementary Table S1). Because some interaction strata contained relatively small numbers, these analyses should be considered exploratory.

Descriptive analyses according to initial symptom category are provided in Supplementary Table S2. Presentation delay differed substantially across symptom categories (*p* < 0.001), ranging from 49.4% among patients presenting with a breast lump to 87.3% among those reporting a long-standing breast lump. In contrast, the proportion with stage III–IV disease did not differ significantly across initial symptom categories (*p* = 0.155).

In sensitivity analysis using a more stringent threshold of ≥4 months, 102 of 244 patients (41.8%) met the definition of presentation delay. The findings were consistent with the primary analysis. Compared with breast lump, long-standing breast lump was associated with higher adjusted odds of delay (aOR 10.84, 95% CI 4.82–24.39; *p* < 0.001), as was breast pain (aOR 2.87, 95% CI 1.42–5.82; *p* = 0.003) and nipple changes (aOR 3.80, 95% CI 1.43–10.09; *p* = 0.007) (Supplementary Table S3).

## 4. Discussion

This study provides contemporary evidence on presentation delay among symptomatic women with breast cancer in Mongolia. Four principal findings emerged. First, presentation delay was common: 69.3% of symptomatic patients waited ≥1 month after recognizing their initial breast symptom before seeking medical consultation. Second, among the factors measured in this study, initial presenting symptom showed the strongest association with presentation delay. Compared with women presenting with a breast lump, those reporting a long-standing breast lump, breast pain, or nipple changes had substantially greater odds of delayed presentation. Third, age, marital status, education, residence, and BMI were not independently associated with presentation delay. Finally, presentation delay was strongly associated with advanced-stage disease at diagnosis: women with a delay of ≥1 month had approximately 4.5-fold higher adjusted odds of stage III–IV disease. In contrast, the association with tumor size ≥ 5 cm did not reach statistical significance.

The high frequency of presentation delay observed in our study is consistent with evidence from many LMIC settings, although direct comparison requires caution because definitions of delay vary considerably across studies. A systematic review and meta-analysis including 50 studies and 18,014 patients from LMICs demonstrated marked regional variation in patient intervals, with longer intervals generally reported in the Middle East, Southeast Asia, and Africa [14]. A more recent systematic review similarly found that the interval from symptom recognition to diagnosis was frequently prolonged and generally contributed more to overall delay than the subsequent treatment interval [15]. In Ethiopia, a 2024 systematic review and meta-analysis estimated that 58.5% of women with breast cancer delayed seeking healthcare, with a median delay of 120 days [16]. Studies from China have likewise identified the patient interval as an important component of overall breast cancer delay [17]. These findings support the broader relevance of delays occurring before patients enter the healthcare system, while differences in thresholds and healthcare contexts preclude simple comparison of prevalence estimates.

The particularly strong association between the initial breast symptom and presentation delay is one of the most important findings of the present study. Compared with women whose initial symptom was a breast lump, those reporting a long-standing breast lump had almost eightfold greater adjusted odds of delay, while breast pain and nipple changes were associated with approximately fourfold and threefold greater odds, respectively. These findings are consistent with evidence that women do not respond uniformly to different breast symptoms. Koo et al. reported that women presenting with non-lump symptoms were more likely to wait ≥90 days before seeking medical attention than those presenting with a breast lump alone [10]. Population-based evidence from Australia has similarly shown longer diagnostic intervals among women with non-lump symptoms [18]. Together, these observations suggest that the way a breast change is recognized and interpreted may be at least as important as the presence of the symptom itself.

The association with long-standing breast lumps deserves particular attention. In the source data, this category represented lumps that were initially small or painless and subsequently became more noticeable, including enlargement or the development of pain. Such a pattern suggests that some women may initially interpret a painless or small breast lump as benign or insufficiently serious to require medical attention. Similar observations have been reported in studies from LMIC settings, where symptom appraisal, failure to attribute breast changes to cancer, fear, misconceptions, and waiting for symptoms to progress have been identified as contributors to prolonged patient intervals [14,15,16,19]. This finding has practical relevance because it suggests that breast health messages should emphasize that a new breast lump warrants medical assessment even when it is small, painless, or otherwise not troublesome. However, because symptom histories were obtained retrospectively, the temporal ordering of lump progression, development of pain, and the decision to seek medical consultation could not be established. Therefore, the observed associations should not be interpreted as demonstrating that symptom progression or pain caused patients to delay consultation.

Our findings concerning breast pain are also noteworthy. Breast pain is common and is usually caused by benign conditions, which may reduce its perceived urgency. Nevertheless, in the present study, women whose initial presenting symptom was breast pain had significantly greater odds of presentation delay than those presenting with a breast lump. The broader literature demonstrates that breast cancer can present with a range of non-lump symptoms and that such presentations may be associated with longer help-seeking intervals [10,18]. Public education should therefore avoid implying that a painless lump is the only breast change requiring assessment. At the same time, these findings should not be interpreted to suggest that breast pain itself is highly predictive of malignancy; rather, among women subsequently diagnosed with breast cancer, symptom type was associated with differences in help-seeking behavior.

In contrast to symptom characteristics, none of the sociodemographic variables examined—age, marital status, education, residence, or BMI—was independently associated with presentation delay. This differs partly from international evidence. The meta-analysis by Petrova et al. identified older age, unmarried status, lower socioeconomic position, illiteracy, limited cancer knowledge, and low social support among factors associated with longer patient intervals in LMICs [14]. Similarly, recent evidence from Ethiopia identified rural residence, lower educational attainment, and longer travel distances as important determinants of delayed presentation [16]. However, systematic evidence also demonstrates substantial heterogeneity in sociodemographic associations across settings [12]. Our results therefore reinforce the importance of evaluating determinants locally rather than assuming that risk factors identified in one population will necessarily apply in another. In our study, patients aged <50 years constituted a somewhat larger proportion of the ≥1-month delay group than of the <1-month group; however, age was not independently associated with presentation delay. This differs from the overall pattern reported by Petrova et al., in which older age was associated with longer patient intervals across several LMIC settings, and may reflect differences in population characteristics, healthcare context, or unmeasured determinants of help-seeking [14].

The absence of an independent association with residence is particularly relevant in Mongolia because specialist cancer services are highly centralized and patients outside Ulaanbaatar may face considerable geographical barriers [12]. However, residence categorized simply as Ulaanbaatar versus outside Ulaanbaatar may not adequately capture healthcare accessibility. Distance to the nearest healthcare facility, travel time, transportation availability, referral pathways, socioeconomic resources, and the location where patients initially sought care were not incorporated into the present model. International evidence indicates that geographical distance and healthcare accessibility can substantially influence breast cancer presentation and diagnostic intervals [15,16]. Thus, our finding should not be interpreted as evidence that geographical access is unimportant in Mongolia; rather, more detailed measures of access may be needed in future studies.

Another major finding was the strong relationship between presentation delay and disease stage. Stage III–IV disease was present in 39.1% of patients with presentation delay compared with 13.3% of those presenting within one month. After adjustment for age, residence, and education, presentation delay was associated with a 4.48-fold increase in the odds of stage III–IV disease. This is consistent with accumulating evidence linking prolonged patient intervals with advanced-stage breast cancer. A recent meta-analysis from Ethiopia identified delay in seeking care as a determinant of advanced-stage diagnosis [20], while broader evidence from Africa has similarly linked delayed presentation and limited breast cancer awareness with late-stage disease [12]. These findings are especially relevant to Mongolia, where late-stage breast cancer has historically been identified as an important challenge [12].

Nevertheless, the association between presentation delay and advanced stage should be interpreted cautiously. Our observational design cannot establish that delayed presentation caused advanced-stage disease. More aggressive tumors may progress rapidly despite relatively short patient intervals, whereas biologically indolent tumors may remain at an earlier stage despite longer intervals. Subsequent diagnostic and healthcare-system delays may also contribute to stage at diagnosis. The absence of a statistically significant association between presentation delay and tumor size ≥ 5 cm in our study further illustrates that the relationship between time and tumor characteristics is complex. Accordingly, our findings demonstrate an association between presentation delay and advanced stage rather than a direct causal effect.

Inflammatory breast cancer is also relevant when considering symptomatic presentation because it may present with rapidly developing inflammatory breast changes rather than a distinct breast lump and is frequently diagnosed at an advanced stage. Its clinical presentation can create particular diagnostic challenges, emphasizing the importance of recognizing the broader spectrum of breast cancer symptoms. However, inflammatory breast cancer was not separately identified in our dataset; therefore, its specific association with presentation delay could not be evaluated in the present study [21].

The findings have several implications for breast cancer early-diagnosis strategies in Mongolia. Public awareness interventions may benefit from moving beyond messages centered exclusively on the classic breast lump. Women should be encouraged to seek assessment for persistent or changing breast abnormalities, including nipple changes and other concerning symptoms, and should understand that a breast lump does not need to be painful or large to warrant evaluation. This approach is consistent with international recommendations emphasizing breast health awareness, timely evaluation of symptomatic women, strengthened primary-care recognition, and efficient referral pathways [4,5,15]. Importantly, our results suggest that interventions based solely on broad sociodemographic targeting may overlook symptom-related differences in help-seeking behavior.

The study also has implications for healthcare professionals. Improving public awareness alone may be insufficient if timely presentation is not followed by prompt clinical assessment and referral. Strengthening the ability of primary healthcare providers to recognize potentially suspicious breast symptoms and ensuring efficient pathways to diagnostic imaging, pathological confirmation, and specialist care are complementary components of early diagnosis. The 2024 systematic review of breast cancer pathways in LMICs emphasized public awareness, healthcare professional capacity, decentralization of diagnostic services, and effective referral mechanisms as interconnected strategies for reducing delays [15]. These considerations may be particularly pertinent in Mongolia because of its dispersed population and centralized specialist oncology services.

This study has several strengths. By restricting the primary analysis to women with a patient-recognized breast symptom, the study specifically evaluates the interval relevant to symptomatic help-seeking and avoids combining symptom-detected cancers with cancers identified through screening or incidental examination. The original dataset included women diagnosed during early-detection or other examinations and women reporting no noticeable breast changes, who were excluded from the present primary analysis. The study also evaluated both determinants of presentation delay and its relationship with tumor characteristics, allowing the potential clinical relevance of delayed presentation to be examined. Several limitations should be acknowledged. First, the retrospective measurement of the patient interval may be subject to both recall and documentation bias. The timing of initial symptom recognition was based on patient-reported information documented in medical and study records and may therefore have been recalled imprecisely or recorded inconsistently. This concern is particularly relevant for patients reporting very long intervals. Because presentation delay was defined directly from the recorded interval between symptom recognition and first medical consultation, measurement error could have resulted in misclassification around the ≥1-month threshold. Second, the study was conducted at a tertiary cancer center and included patients who reached specialist care; therefore, the findings may not fully represent women with breast cancer who were never referred, were treated elsewhere, or faced barriers preventing access to cancer services. Third, several potentially important determinants of help-seeking—including breast cancer knowledge, symptom interpretation, fear of diagnosis, financial constraints, use of traditional or alternative medicine, travel distance, social support, and detailed healthcare accessibility—were not available and could not be included in the multivariable model. In particular, residence was categorized only as Ulaanbaatar versus outside Ulaanbaatar and therefore represents a crude proxy for geographic access. This measure does not capture potentially important differences in travel distance, transportation availability, seasonal or weather-related barriers, referral pathways, local primary-care capacity, availability of diagnostic imaging, or the ability to travel to the National Cancer Center. Consequently, the absence of an association between residence and presentation delay should not be interpreted as evidence that geographic access is unimportant. These factors have been associated with patient intervals in previous studies [14,15,16]. Therefore, the strong association observed for initial presenting symptom should be interpreted in the context of the variables available in this study and should not be considered evidence that symptom type is the dominant determinant of help-seeking behavior in the broader Mongolian population.

Fourth, the ≥1-month threshold provides a clinically interpretable definition for the primary analysis but represents a categorization of an inherently continuous time interval, and thresholds used in previous studies vary substantially. Fifth, some symptom categories contained relatively small numbers, resulting in relatively wide confidence intervals, particularly for the long-standing breast lump and nipple-change groups. Finally, because of the observational design, the association between presentation delay and advanced-stage disease should not be interpreted as causal. Despite these limitations, the study provides context-specific evidence on symptomatic breast cancer presentation in Mongolia and identifies potentially actionable areas for earlier diagnosis.

Future prospective studies should collect standardized dates for symptom recognition, first healthcare consultation, referral, diagnostic confirmation, and treatment initiation in accordance with established frameworks for cancer diagnostic intervals. Such studies should additionally assess breast cancer knowledge, symptom appraisal, psychological barriers, financial constraints, healthcare utilization, travel distance, and referral pathways. Larger multicenter studies incorporating both Ulaanbaatar and regional healthcare facilities would help determine whether the observed associations are consistent across Mongolia and would allow patient-related and healthcare-system delays to be evaluated separately.

## 5. Conclusions

Presentation delay was common among symptomatic breast cancer patients in Mongolia, with more than two-thirds seeking medical consultation ≥1 month after recognizing their initial breast symptom. Among the factors measured in this study, initial presenting symptom showed the strongest association with presentation delay, whereas age, marital status, education, residence, and BMI were not independently associated with delay. Patients reporting a long-standing breast lump, breast pain, or nipple changes had higher odds of presentation delay than those presenting with a breast lump. Presentation delay was also associated with higher odds of stage III–IV disease at diagnosis. These findings support breast health education that emphasizes timely medical evaluation of a broad range of breast symptoms, including small or painless lumps and non-lump changes. Prospective and multicenter studies are needed to further examine patient and healthcare-system factors contributing to delayed breast cancer diagnosis in Mongolia.

## Figures and Tables

**Figure 1:**
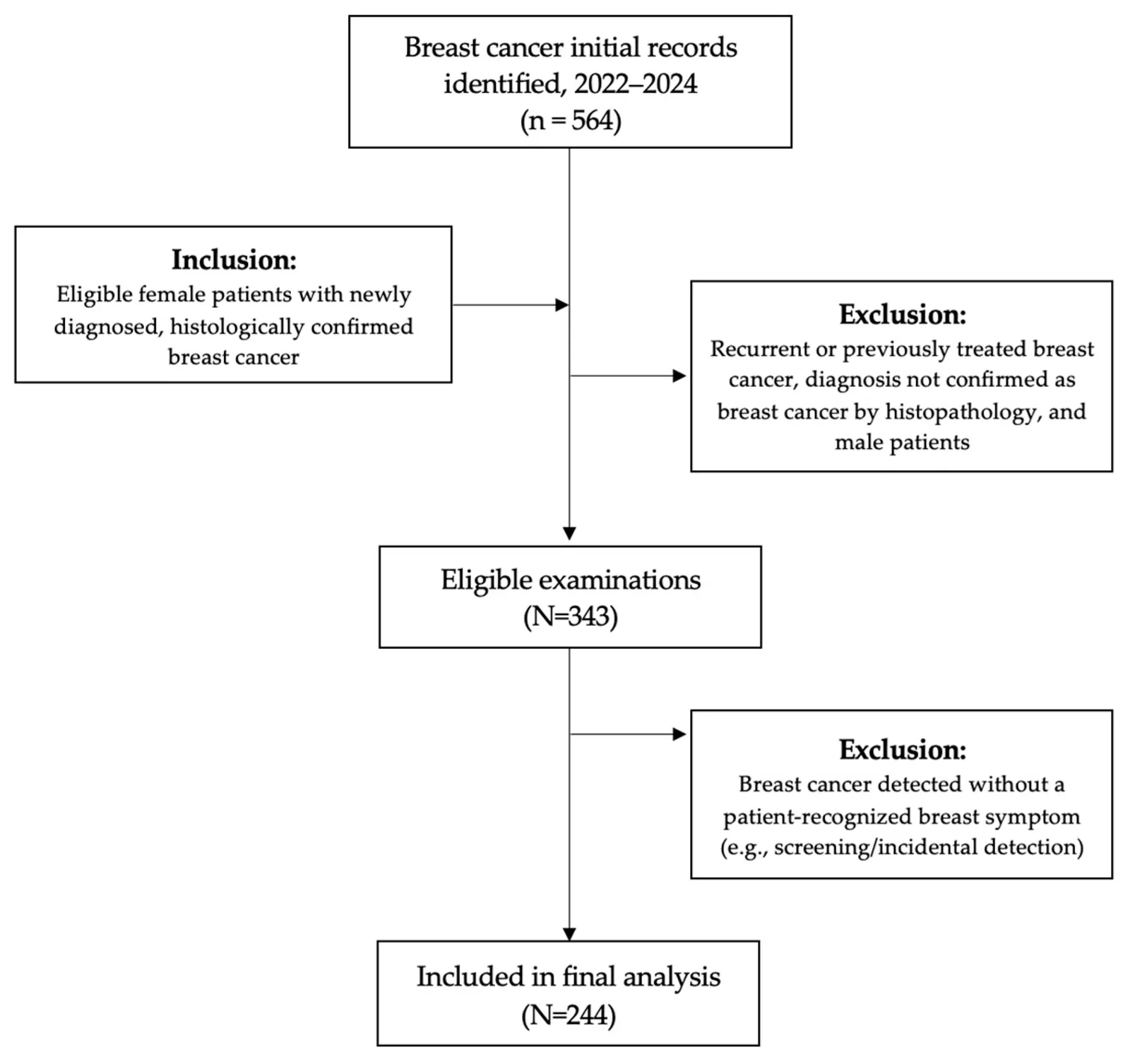
Flow diagram of participant selection for the study.

**Figure 2:**
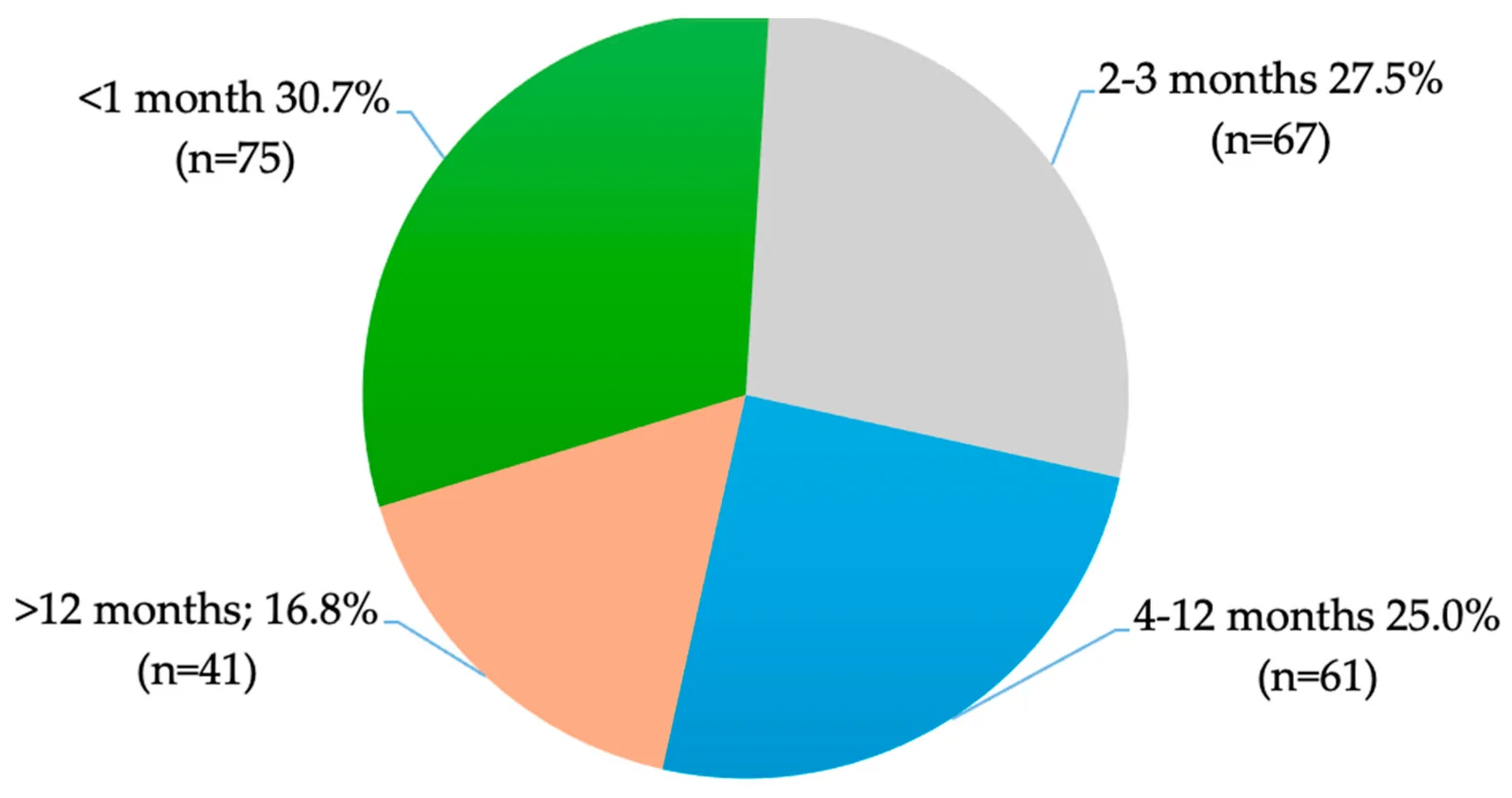
Distribution of time from initial breast symptom recognition to first medical consultation among symptomatic breast cancer patients.

**Table 1:** Participant characteristics according to presentation delay among symptomatic breast cancer patients.

Characteristic	Overall N = 244, n (%)	<1 Month N = 75, n (%)	≥1 Month N = 169, n (%)	*p*-Value
Age group, years				0.424
<50	102 (41.8)	28 (37.3)	74 (43.8)	
50–59	80 (32.8)	24 (32.0)	56 (33.1)	
≥60	62 (25.4)	23 (30.7)	39 (23.1)	
Marital status				0.593
Married	190 (77.9)	60 (80.0)	130 (76.9)	
Not married/no husband	54 (22.1)	15 (20.0)	39 (23.1)	
Education level				0.864
Secondary or lower	90 (36.9)	26 (34.7)	64 (37.9)	
Vocational/technical	42 (17.2)	14 (18.7)	28 (16.6)	
University or higher	112 (45.9)	35 (46.7)	77 (45.6)	
Residence				0.597
Ulaanbaatar	137 (56.1)	44 (58.7)	93 (55.0)	
Outside Ulaanbaatar	107 (43.9)	31 (41.3)	76 (45.0)	
BMI category				0.743
<25 kg/m^2^	85 (34.8)	25 (33.3)	60 (35.5)	
≥25 kg/m^2^	159 (65.2)	50 (66.7)	109 (64.5)	

Notes: Data are n (%). Percentages in the <1 month and ≥1 month columns are column percentages. Presentation delay was defined as ≥1 month from recognition of the initial breast symptom to first medical consultation. *p*-values are from Pearson’s χ^2^ tests. BMI, body mass index.

**Table 2:** Clinical and tumor characteristics according to presentation delay among symptomatic breast cancer patients.

Characteristic	Overall N = 244, n (%)	<1 Month N = 75, n (%)	≥1 Month N = 169, n (%)	*p*-Value
Initial presenting breast symptom				<0.001
Breast lump	85 (34.8)	43 (57.3)	42 (24.9)	
Long-standing breast lump	55 (22.5)	7 (9.3)	48 (28.4)	
Breast pain	79 (32.4)	18 (24.0)	61 (36.1)	
Nipple changes	25 (10.2)	7 (9.3)	18 (10.7)	
Tumor size				0.073
<5 cm	218 (89.3)	71 (94.7)	147 (87.0)	
≥5 cm	26 (10.7)	4 (5.3)	22 (13.0)	
Cancer stage				0.003
In situ	11 (4.5)	4 (5.3)	7 (4.1)	
Stage I	54 (22.1)	20 (26.7)	34 (20.1)	
Stage II	103 (42.2)	41 (54.7)	62 (36.7)	
Stage III	51 (20.9)	7 (9.3)	44 (26.0)	
Stage IV	25 (10.2)	3 (4.0)	22 (13.0)	
Histological type				0.749
Ductal carcinoma	168 (68.9)	51 (68.0)	117 (69.2)	
Lobular carcinoma	30 (12.3)	9 (12.0)	21 (12.4)	
Other histology	35 (14.3)	10 (13.3)	25 (14.8)	
Breast carcinoma/other coded category	11 (4.5)	5 (6.7)	6 (3.6)	
Molecular/IHC subtype				0.887
Luminal A	68 (27.9)	24 (32.0)	44 (26.0)	
Luminal B	65 (26.6)	20 (26.7)	45 (26.6)	
HER2+	29 (11.9)	8 (10.7)	21 (12.4)	
Triple-negative breast cancer	34 (13.9)	9 (12.0)	25 (14.8)	
IHC code 0/unspecified	48 (19.7)	14 (18.7)	34 (20.1)	

Notes: Data are n (%). Percentages in the <1 month and ≥1 month columns are column percentages. Presentation delay was defined as ≥1 month from recognition of the initial breast symptom to first medical consultation. *p*-values are from Pearson’s χ^2^ tests. HER2, human epidermal growth factor receptor 2; IHC, immunohistochemistry.

**Table 3:** Factors associated with presentation delay ≥1 month among symptomatic breast cancer patients.

Characteristic	Crude OR (95% CI)	*p*-Value	Adjusted OR (95% CI) *	*p*-Value
Age group, years				0.185 †
<50	1.00 (Reference)	-	1.00 (Reference)	-
50–59	0.88 (0.46–1.69)	0.706	0.94 (0.46–1.94)	0.867
≥60	0.64 (0.33–1.26)	0.197	0.52 (0.24–1.10)	0.088
Marital status				
Married	1.00 (Reference)	-	1.00 (Reference)	-
Not married/no husband	1.20 (0.61–2.34)	0.593	1.22 (0.59–2.54)	0.590
Education level				0.770 †
Secondary or lower	1.00 (Reference)	-	1.00 (Reference)	-
Vocational/technical	0.81 (0.37–1.78)	0.605	0.82 (0.35–1.90)	0.635
University or higher	0.89 (0.49–1.64)	0.716	1.12 (0.56–2.24)	0.753
Residence				
Ulaanbaatar	1.00 (Reference)	-	1.00 (Reference)	-
Outside Ulaanbaatar	1.16 (0.67–2.01)	0.597	1.15 (0.62–2.15)	0.655
BMI category				
<25 kg/m^2^	1.00 (Reference)	-	1.00 (Reference)	-
≥25 kg/m^2^	0.91 (0.51–1.61)	0.743	0.97 (0.52–1.81)	0.917
Initial presenting breast symptom		<0.001 †		<0.001 †
Breast lump	1.00 (Reference)	-	1.00 (Reference)	-
Long-standing breast lump	7.02 (2.85–17.26)	<0.001	7.92 (3.14–19.98)	<0.001
Breast pain	3.47 (1.76–6.82)	<0.001	3.80 (1.89–7.66)	<0.001
Nipple changes	2.63 (1.00–6.95)	0.051	3.32 (1.20–9.21)	0.021

Notes: OR, odds ratio; CI, confidence interval; BMI, body mass index. The outcome was presentation delay ≥1 month; presentation within <1 month was the reference outcome. * Adjusted estimates are mutually adjusted for age group, marital status, education, residence, BMI, and initial presenting symptom. † Overall Wald *p*-value for the categorical variable.

**Table 4:** Association of presentation delay with tumor characteristics at diagnosis among symptomatic breast cancer patients.

Tumor Characteristic	Crude OR (95% CI)	*p*-Value	Adjusted OR * (95% CI)	*p*-Value
Tumor size ≥ 5 cm	2.66 (0.88–8.00)	0.082	2.59 (0.83–8.08)	0.100
Stage III–IV	4.17 (2.00–8.68)	<0.001	4.48 (2.12–9.49)	<0.001

* Adjusted for age, residence, and education. ORs compare patients with presentation delay ≥1 month with those presenting within <1 month.

## Data Availability

The data supporting the findings of this study are available from the corresponding author upon reasonable request. The data are not publicly available due to patient privacy and ethical restrictions.

## Supplementary Materials

The following supporting information can be downloaded at: https://www.mdpi.com/article/10.3390/healthcare14193270/s1, Table S1: Exploratory interaction analyses for presentation delay; Table S2: Presentation delay and advanced-stage disease according to initial presenting symptom. Table S3: Sensitivity analysis of factors associated with presentation delay using a ≥4-month threshold.
