# Supplementary material for: Presentation Delay After Symptom Recognition Among Breast Cancer Patients in Mongolia: A Retrospective Study
Source: Healthcare (Basel). 2026 Oct 2;14(19):3270. doi: 10.3390/healthcare14193270 (PMC13664980; doi:10.3390/healthcare14193270)
Supplement: Supplementary file 1 [file healthcare-14-03270-s001.zip › healthcare-4552482-supplementary.pdf]

healthcare

MDPI

Supplementary Table S1. Exploratory interaction analyses for presentation delay

Interaction tested	χ^2	df	p-value	Interpretation
Initial symptom × residence	2.692	3	0.442	No statistically significant interaction
Initial symptom × education	3.547	6	0.738	No statistically significant interaction

Note: Interaction terms were evaluated separately in multivariable logistic regression models for presentation delay ≥1 month. Models included the corresponding main effects and adjustment covariates. Because some interaction strata contained relatively small numbers, individual interaction coefficients should not be emphasized.

Supplementary Table S2. Presentation delay and advanced-stage disease according to initial presenting symptom

Initial presenting symptom	N	Presentation delay ≥1 month, n (%)	Stage III–IV, n (%)
Breast lump	85	42 (49.4)	20 (23.5)
Long-standing breast lump	55	48 (87.3)	22 (40.0)
Breast pain	79	61 (77.2)	24 (30.4)
Nipple changes	25	18 (72.0)	10 (40.0)
**Total**	**244**	**169 (69.3)**	**76 (31.1)**
**p-value**		**<0.001**	**0.155**

Note: Percentages are calculated within each initial symptom category. Presentation delay was defined as ≥1 month from recognition of the initial breast symptom to first medical consultation. Advanced-stage disease was defined as stage III–IV. P-values are from Pearson's χ^2 tests.

Supplementary Table S3. Sensitivity analysis of factors associated with presentation delay using a ≥4-month threshold

Initial presenting symptom	≥4-month delay, n/N (%)	Crude OR (95% CI)	Adjusted OR (95% CI)*	p-value
Breast lump	17/85 (20.0)	1.00	1.00	Reference
Long-standing breast lump	40/55 (72.7)	10.67 (4.81–23.66)	**10.84 (4.82–24.39)**	<0.001
Breast pain	33/79 (41.8)	2.87 (1.43–5.75)	**2.87 (1.42–5.82)**	0.003
Nipple changes	12/25 (48.0)	3.69 (1.43–9.53)	**3.80 (1.43–10.09)**	0.007

Note: *Adjusted for age group, marital status, education, residence, overweight/obesity, and initial presenting symptom.
